# Supplementary material for: Osr1 Is Required for Mesenchymal Derivatives That Produce Collagen in the Bladder
Source: Int J Mol Sci. 2021 Nov 17;22(22):12387. doi: 10.3390/ijms222212387 (PMC8619163; doi:10.3390/ijms222212387)
Supplement: Supplementary file 1 [file ijms-22-12387-s001.zip › ijms-1415111-supplementary.pdf]

Supplementary Material

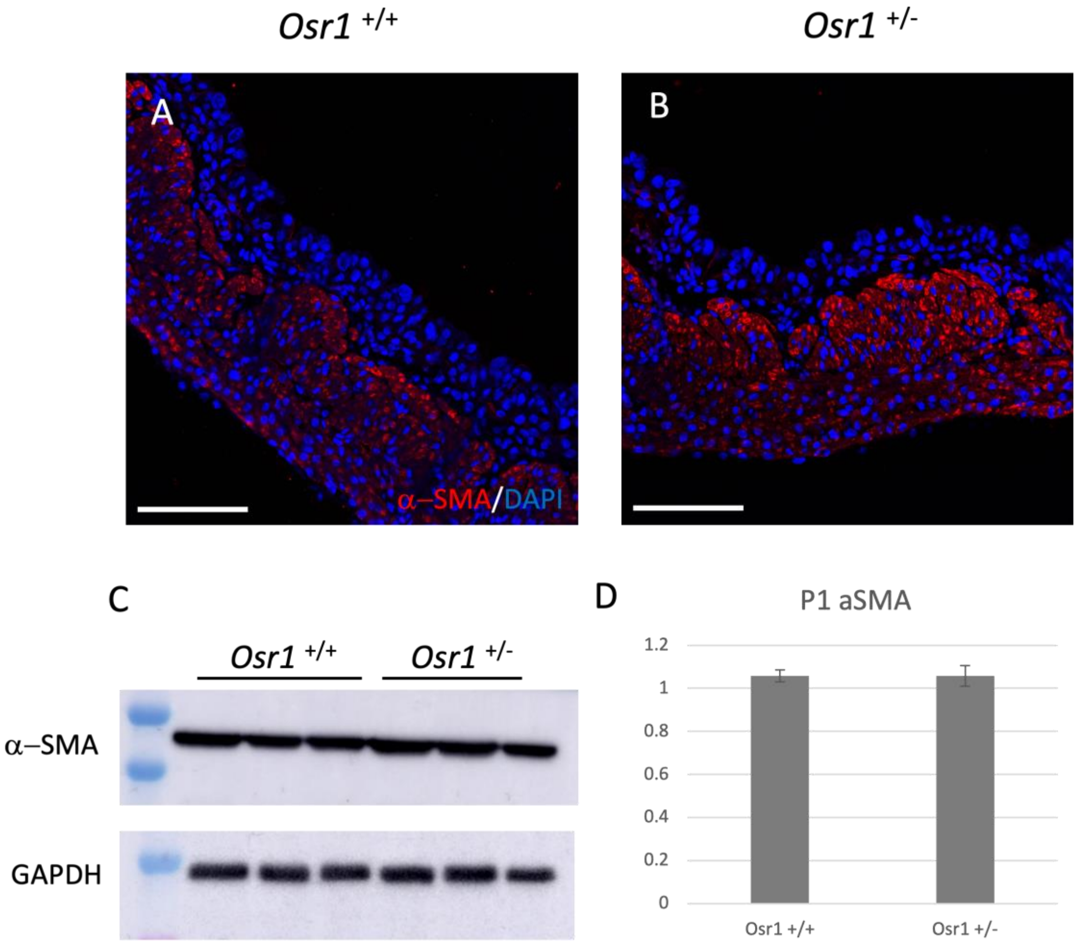

Figure S1. Osr1$^{+/−}$ newborn mouse bladders have similar levels of α-SMA: Immunofluorescent staining for α-SMA in red, and DAPI in blue shows no difference in the bladders between *Osr1*$^{+/+}$ (**A**) and *Osr1*$^{+/−}$ (**B**) newborn mice. (**C,D**) No difference was seen on western blot analysis when normalized to GAPDH. Scale bar = 100 μm. N = 3 mice/genotype.

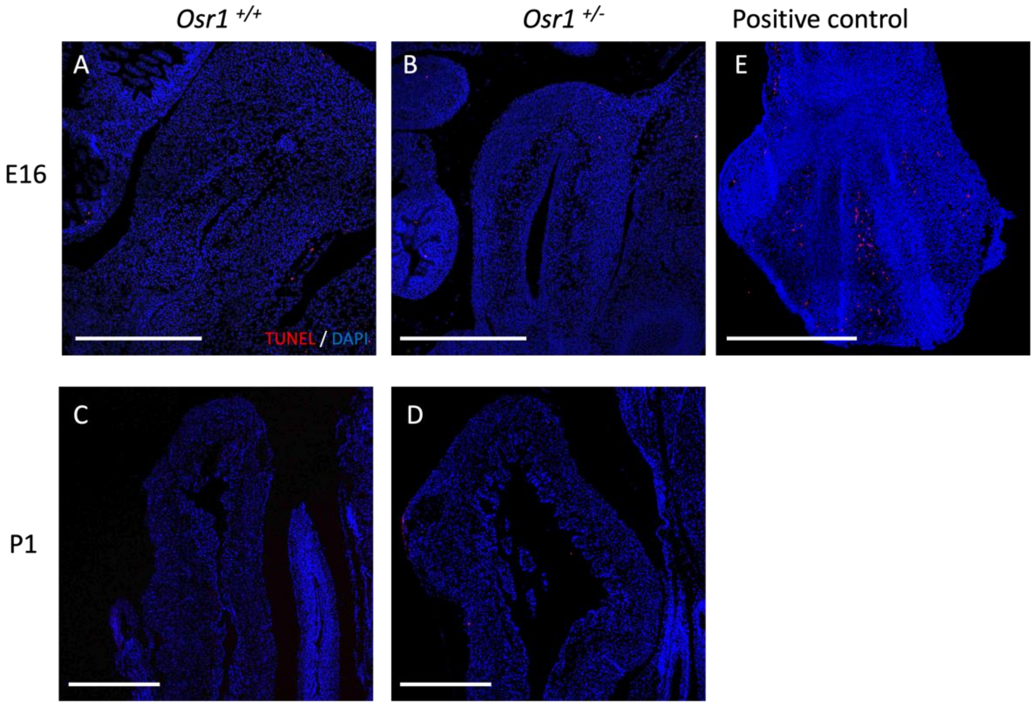

Figure S2. Little evidence of apoptosis seen by TUNEL staining at E16 or P1: Fluorescent imaging of the TUNEL assay showed no cell death in bladders in *Osr1*$^{+/+}$ or *Osr1*$^{+/-}$ embryos at E16 (**A,B**) or pups at P1 (**C,D**). (**E**) Interdigital region of E12 mouse limb is shown as positive control. Scale bar = 500 μm.

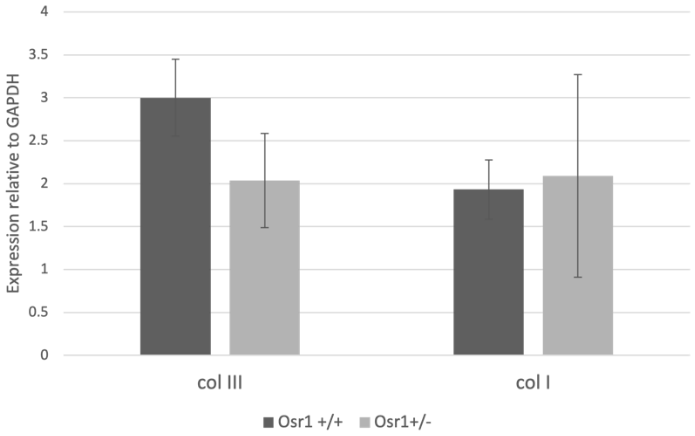

Figure S3. No difference in transcript levels of *col III* or *col I* between Osr1$^{+/+}$ **or *Osr1***$^{+/-}$ **mouse bladders:** Bar graphs representing expression levels of *col III* and *col I* relative to gapdh shows no difference in mRNA expression levels between *Osr1*$^{+/+}$ or *Osr1*$^{+/-}$ mice (*col III: Osr1*$^{+/+}$ 3.0014+/– 0.45 vs. *Osr1*$^{+/-}$ 2.03+/– 0.34, $p = 0.08$, *col I: Osr1*$^{+/+}$ 1.93+/- 0.54 vs. *Osr1*$^{+/-}$ 2.09+/– 1.17, $p = 0.87$).

Table S1. Comparison between cystometry parameters in Osr1$^{+/+}$ and Osr1$^{+/-}$ male and female mice

	Wild type		OSR1 $^{+/-}$		*p* value*
	Female	Male	Female	Male	
**Basal pressure**	12.98 (7.36)	9.86 (5.75)	11.63 (9.72)	15.20 (9.10)	0.719
**Intermicturition pressure**	27.84 (11.29)	24.27 (5.46)	20.08 (20.31)	26.87 (5.92)	0.244
**Threshold pressure**	47.21 (13.38)	42.67 (11.65)	38.84 (26.67)	48.61 (14.65)	0.733
**Maximum pressure**	117.58 (39.19)	103.73 (26.87)	74.33 (48.49)	116.03 (50.47)	0.385
**Micturition volume**	0.071 (0.043)	0.060 (0.023)	0.030 (0.005)	0.034 (0.008)	**0.01***
**Intercontraction interval**	169.50 (68.85)	160.62 (42.0)	86.63 (31.69)	78.76 (23.53)	**0.005***
**Spontaneous activity**	14.86 (4.46)	14.41 (5.97)	8.45 (10.90)	11.66 (8.70)	0.199
**Bladder capacity**	0.080 (0.033)	0.071 (0.017)	0.037 (0.013)	0.036 (0.011)	**0.001***
**Residual volume**	0.013 (0.008)	0.013 (0.008)	0.010 (0.010)	0.004 (0.005)	0.276
**Bladder compliance**	0.003 (0.001)	0.003 (0.002)	0.002 (0.002)	0.001 (0.000)	**0.031***

Data are presented as mean (SD)
**p* value for a difference between the 4 groups using Kruskal-Wallis test.
